# Behavioral difficulties and associated factors among the ‘lost generation’ of Syrian children and adolescents

**DOI:** 10.1038/s41598-024-59784-z

**Published:** 2024-04-23

**Authors:** Aya Alsharif, Osama Al Habbal, Aram Gabadian, Riwa El Maamoun, Alaa Al Faraj, Taima kamr aldin, Ola Haitham Aldammad, Ahmad Alkayakhi, Aya Al Habbal

**Affiliations:** 1 Faculty of Medicine, Damascus University, Damascus, Syria; 2 Eye Surgical Hospital, Damascus, Syria

**Keywords:** Behavioral difficulties, Strength and difficulties questionnaire, WHO quality of life questionnaire, Syrian crises, Syrian children, Neuroscience, Psychology, Medical research

## Abstract

Childhood and adolescence, vital in shaping adult life and society, are profoundly impacted during conflicts like Syria’s devastating war. This study explores the prevalence of behavioral disorders in Syrian children and adolescents, examining the influence of war and family-related factors. This cross-sectional study was conducted on children aged 2–17 years at a children’s outpatient clinic in Damascus, Syria. We assessed parents’ quality of life, war and family-related factors, and behavioral difficulties through parental interviews using two questionnaires: the Arabic version of the Strengths & Difficulties Questionnaire (SDQ) and the brief Arabic version of the World Health Organization Quality of Life Questionnaire (WHOQOL-BREF). 74.67% of children aged 2–4 years and 61.29% of children aged 5–17 years were experiencing behavioral difficulties, with emotional difficulties being the most prevalent ones. Children exposed to kidnapping, family losses, lack of school enrollment, and those with parents having lower education, lower socioeconomic status, and poorer quality of life exhibited higher Total SDQ scores. The high prevalence of behavioral difficulties among children and adolescents in Syria is a major concern, with both direct and indirect war-related factors contributing to this issue.

## Introduction

Human life is a fleeting journey, beginning in childhood, a phase when one remains largely unaware of its profound significance in shaping the trajectory of their life^1^, their immediate society, and even their nation^2,3^. This early phase could chart the course of one's adolescence, a time marked by substantial development across emotional, biological, cognitive, and psychosocial domains^4,5^, ultimately influencing their transition into adulthood^2^. Consider, for instance, that the education a child receives during their formative years can determine the extent of their schooling and, in turn, impact their income, which ripples through the entire nation's economy^6^. Adolescence renders individuals particularly susceptible to behavioral and mental health challenges, even when exposed to seemingly minor stressors like stringent parenting and school interactions^7^. Now, when we consider the implications of stress in conflict regions like Syria, we begin to fathom the dire circumstances.

The Syrian war is one of the most devastating humanitarian catastrophes and extensive displacement crises of the 21st century^8,9^. The Syrian conflict persists with continuous hostilities, leading to a significant loss of life, extensive civilian infrastructure destruction, and the erosion of the health system and social services^9,10^. Concurrently, the country is suffering from an escalating economic crisis characterized by high inflation rates, fuel scarcities, and currency devaluation^11,12^, pushing nearly 90% of the population below the poverty line^13^. As the conflict has endured for eleven years, compounded by two years of the COVID-19 pandemic and the worsening economic crisis, children in Syria bear the brunt of one of the most intricate and dire humanitarian emergencies worldwide^14,15^. An entire generation of children, approximately 6 million born since the war’s onset in 2011, has known nothing but a life entrenched in conflict and displacement^14^. At present, approximately 6.9 million children need immediate humanitarian assistance^15^. Disturbingly, in 2020, 2.45 million children were not receiving formal education, a number presumed to have risen since^13^. These dire circumstances have taken a toll on children's psychological well-being, with one in three children in Syria exhibiting signs of psychological distress in 2021. Regrettably, in this context, an estimated 75% of individuals grappling with mental health issues do not have access to any form of treatment^16,17^. Syrian children, the potential bearers of a brighter future, emerge as among the most vulnerable victims of this protracted conflict. To prevent the risk of a lost generation, organizations and governments must prioritize enhancing the mental well-being of the millions of Syrian children, who have endured a decade of trauma and loss, as they represent the crucial human capital necessary for Syria's recovery, reconstruction, and progress once the conflict comes to an end^13^. Monitoring the prevalence of emotional and behavioral issues in children is the initial step in determining their magnitude^18^.

The majority of existing studies have focused on the psychological well-being of Syrian refugees in neighboring countries ^13,19–22^, while children within Syria have received little attention. Currently, there is a lack of data concerning the prevalence of emotional and behavioral issues among children and adolescents in Syria. As a result, it is essential to examine the behavioral challenges faced by Syrian children, at the very least, to establish a baseline for understanding the scope of the issue. The objective of this study was to investigate the prevalence of behavioral disorders in children and adolescents residing in Syria and to examine the potential impact of war on children's behavior. Furthermore, the family's role is pivotal in children's well-being, and disruptions stemming from war, displacement, and related stressors can adversely lead to parenting challenges and compromising family communication, often posing risks to children's development^23–26^. Given the family’s significant role in the mental health of Syrian children and adolescents, this study also aims to explore the association between family-related factors, such as parental quality of life, and children’s behavior. As we delve into the complexities of childhood and adolescence in conflict-ridden regions, this study seeks to address a fundamental question: What is the prevalence of behavioral disorders among Syrian children and adolescents, and how are these disorders influenced by war-related factors and family-related variables, including parental quality of life? By exploring these crucial aspects, we aim to contribute valuable insights into the impact of the Syrian conflict on the mental well-being of its youngest generation.

## Material and methods

### Study design

This cross-sectional study was carried out at the pediatric clinics of the Children’s Hospital in Damascus, Syria.

### Inclusion and exclusion criteria

Children between the ages of 2 and 17 who were visiting the general outpatient clinics at the Children's Hospital and did not have any neurological complaints were eligible to participate in the study, provided that one of their parents gave informed consent. Children with severe cognitive or developmental deficiencies were excluded from the study. Due to the unique circumstances at the children's hospital and the practice of patients arriving at the outpatient clinics without prior appointment registration, we encountered challenges in implementing random sampling. Instead, we opted to approach all parents of children present in the outpatient clinic during the study period, seeking their approval for participation. Only those whose parents granted approval were enrolled in the study.

### Data collection

Trained medical students conducted oral interviews with parents to gather demographic information and information related to war-related factors and family-related variables. This included the child's gender, age, number of siblings, and birth order, as well as both parents' age, education level, employment status, and family income. Additionally, the interview covered aspects such as displacement due to war, exposure to direct danger, and specific traumatic events, aligning with the study's objective to understand how war-related factors influence behavioral disorders in Syrian children and adolescents. During these oral interviews, two main questionnaires were administered: the Arabic version of the Strengths & Difficulties Questionnaire (SDQ) and the brief Arabic version of the World Health Organization Quality of Life Questionnaire (WHOQOL-BREF). The inclusion of these questionnaires within the interview format allowed for a comprehensive assessment of behavior, emotions, relationships, and quality of life for both children and their parents. This method was chosen to ensure a direct and thorough exploration of the research question regarding the prevalence of behavioral disorders in Syrian children and adolescents and the influence of war-related and family-related factors. Family income was classified into three categories: inadequate to cover necessary living expenses, sufficient to meet necessary living expenses, and enough to cover more than just the necessary expenses. The educational levels were categorized into six groups: illiteracy, primary (first to sixth grade), lower secondary (seventh to ninth grade), upper secondary (10th–12th grade), bachelor’s or equivalent, and Master’s, Doctorate or equivalent. Other collected data included the child's current grade level, displacement due to war, type of their residence, and total number of individuals living in the same household, as many families were displaced from their homes and lived with others in small houses.

The WHOQOL-BREF was used to evaluate the quality of life of the parents. This tool, developed by the World Health Organization, measures an individual's perception of their quality of life across multiple dimensions. Notably, it does not measure specific symptoms or diseases^27^. Consisting of 26 questions, the WHOQOL-BREF assesses respondents’ overall quality of life and health as well as their satisfaction over the previous 2 weeks with four main domains: physical health (pain, energy, sleep, mobility, activities of daily living, medication, and work), psychological health (positive feel, thinking, self-esteem, body image, negative feel, and spirituality), social relations (personal relationships, social support), and environment (safety, home environment, financial resources, access to services, access to information, leisure activities, physical environment, and access to transport). Respondents indicate their satisfaction on a 5-point scale ranging from 1 (very poor) to 5 (very good). To calculate the scores, the 4–20 scale is converted to a 0–100% scale^27,28^. To avoid potential discomfort for the participants, the question regarding sexual activities was omitted, as it was deemed inappropriate in the Syrian culture. This exclusion did not affect the validity of the assessment, as only one item was missing. The scores are measured in a positive direction, indicating that higher scores correspond to a higher quality of life^27^. The reliability and validity of the Arabic version of the WHOQOL-BREF are remarkable. Internal consistency values, measured through Cronbach’s alpha, exceeded 0.7 for both the complete questionnaire and its individual domains. Importantly, domain scores exhibited significant discrimination between well and sick groups^28^.

The SDQ is a widely used screening tool to assess the behavior, emotions, and relationships of children and adolescents^29^. It consists of 25 questions that are categorized into 5 areas, including conduct problems, inattention-hyperactivity, emotional symptoms, peer problems, and prosocial behavior^30,31^. Each question is rated on a three-point scale, and the total difficulties score is the sum of all scales except for prosocial behavior because it is considered separate from psychological difficulties^31^. There are two versions of the SDQ, one for children aged 2–4 and another for those aged 4–17. While the two versions share 22 questions, the 2–4 years version has modified questions on reflectiveness and oppositionality instead of antisocial behavior^32^. Children whose scores fall within the abnormal or borderline range are classified as potentially having behavioral disorders^33^. For the 2–4 years version of the SDQ, a slightly raised Total Difficulties Score is within the range of 13–15, indicating mild behavioral difficulties. A high Total Difficulties Score falls within the range of 16–18, indicating significant behavioral difficulties. A very high Total Difficulties Score falls within the range of 19–40, indicating severe behavioral problems. For the 4–17 years version of the SDQ, a slightly raised Total Difficulties Score is within the range of 14–16, while a high Total Difficulties Score falls within the range of 17–19. A very high Total Difficulties Score falls within the range of 20–40. In terms of the Prosocial Behavior Score, for the 2–4 years version, a slightly lowered score is 6, while a low score is 5. A very low score falls within the range of 0–4, indicating little to no prosocial behavior. For the 4–17 years version, a slightly lowered score is 7, while a low score is 6. A very low score falls within the range of 0–5, indicating little to no prosocial behavior^34^. Previous studies have substantiated the psychometric qualities of the SDQ, affirming satisfactory reliability through internal consistency (average Cronbach’s α: 0.73) and establishing validity. The Arabic rendition of the SDQ exhibits sufficient accuracy in predicting psychiatric diagnoses, thereby proving valuable for screening, epidemiological investigations, and clinical assessments. This underscores the SDQ's practicality, cost-effectiveness, and validity, making it a reliable measure for assessing various behavioral facets in children^30,35^. Although the WHOQOL-BREF and SDQ questionnaires are usually self-administered by parents, we opted to gather their responses during the oral interviews. We made this decision considering the social and cultural constraints prevalent in the study population.

In the end, the investigators recorded whether the child had been exposed to direct danger or drug and food shortages resulting from sieges and blockades. They also inquired whether the child had undergone the ordeal of kidnapping or loss of family members, or had witnessed people being injured or killed in bombings and explosions.

### Statistical analysis

The statistical analysis was conducted using R4.2.2 and RStudio. Descriptive statistics were presented with the mean and standard deviation, as well as frequency and percentage. Pearson's correlations were utilized to assess the relationships between normally distributed continuous variables. However, in cases where one of the variables did not follow a normal distribution or was ordinally categorical, Spearman correlations were employed instead. Welch's independent samples t-test was selected over the Student's t-test when comparing the means of two independent continuous groups whose residuals exhibited a normal distribution. This decision was influenced by the better control over the type 1 error provided by Welch's test^36,37^. The Mann–Whitney test was employed if the residuals of these groups did not meet the normal distribution assumption. For comparing the means of three independent groups with the assumption of equal variances and normally distributed residuals, One-way analysis of variance (ANOVA) was conducted. In instances where the observations within these groups did not satisfy the normality or the equal variance assumptions, the Kruskal–Wallis test was chosen. Significance throughout all these statistical tests was defined by a *p*-value of less than 0.05. To address potential familywise errors following significant *p*-values in previous tests, we employed the Holm–Bonferroni Correction for multiple comparisons. In addition, effect size measures, such as Cliff's Delta for Mann–Whitney tests and epsilon for Kruskal–Wallis tests, were included to provide a comprehensive assessment of the magnitude of the observed effects. To assess whether observed differences can be attributed to quality of life and family-related factors or war-related factors, we conducted an analysis of covariance (ANCOVA). Finally, given the presence of multiple predictor variables in our study and to mitigate bias in our analyses, we employed a machine learning model known as conditional random forest^38^. This approach allowed us to identify the most relevant variables in predicting children's behavioral difficulties. By assessing the relative importance of each variable within the model and comparing it to a model built with random noise, we aimed to enhance the accuracy and interpretability of our predictions.

### Ethics

The Ethics Committee of Damascus University approved the study to be conducted, ensuring adherence to relevant guidelines and regulations in the execution of all methods. All parents were required to provide informed consent.

### Ethics approval and consent to participate

Informed consent was taken from children’s parents, and for using and publishing the data. Confidentiality was assured and no data indicative to the person were collected. Our study protocol and ethical aspects were reviewed and approved by Damascus University, Damascus, Syria.

## Results

**Figure 1:**
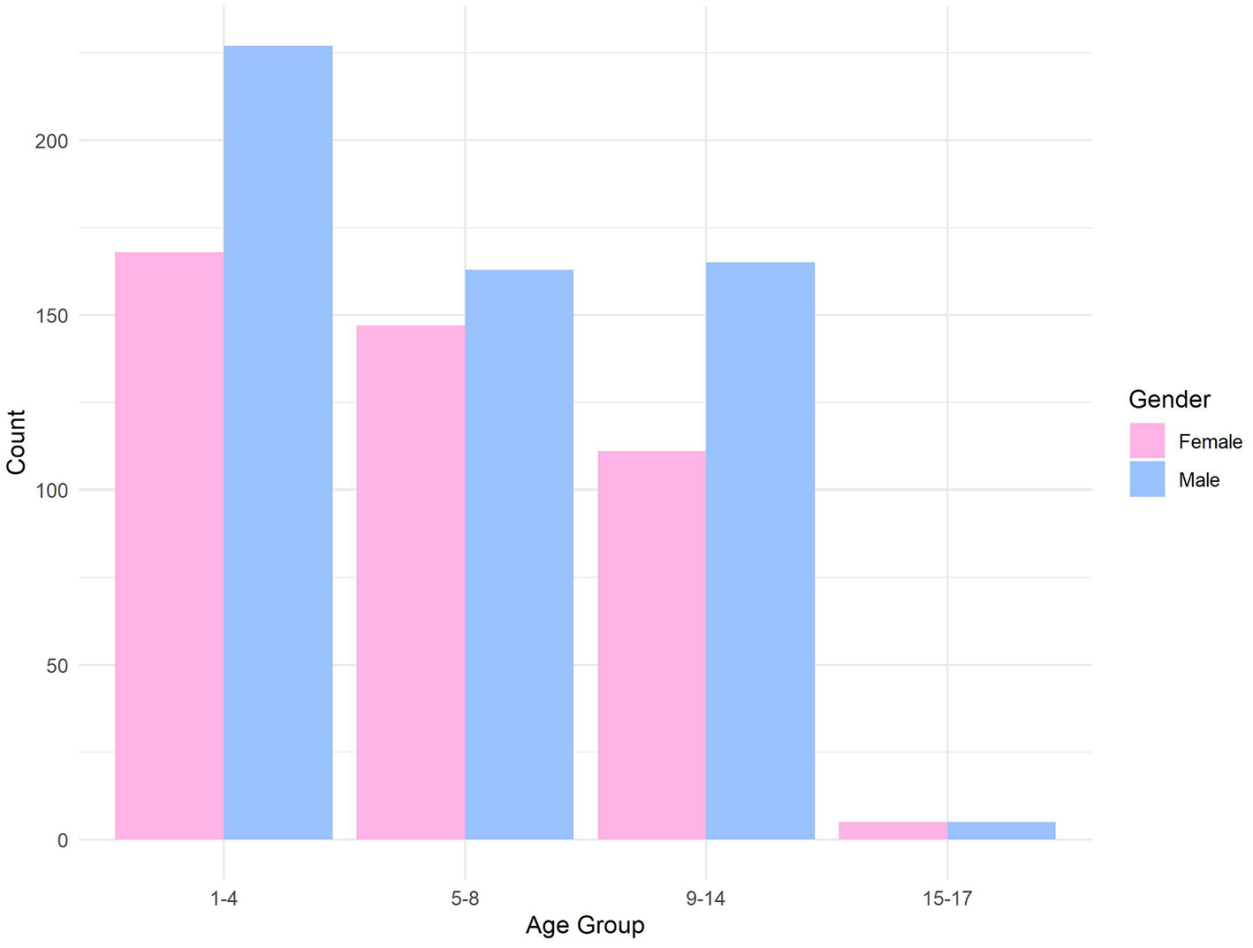
Children's gender distribution across different age groups. Bar chart displaying the distribution of children's gender across different age groups.

This study included 991 children, with a mean age of 7.15 years ± 3.44 and 56.5% being males. The children were categorized into two age groups: 2–4 years old (n = 229) and 5–17 years old (n = 762). The demographics of participants are summarized in Table 1. Figure 1 illustrates the distribution of children across various age groups through a bar chart.

**Table 1:** Demographics of Participants (n = 991).

Age, years	
Mean (SD)	7,15 (3.44)
Mother’s age, years	
Mean (SD)	33.09 (7.39)
Father’s age, years	
Mean (SD)	39.67 (9.03)
Brothers number	
Mean (SD)	1.59 (1.27)
Sisters number	
Mean (SD)	1.62 (1.50)
Total siblings	
Mean (SD)	3.21 (2.13)
Birth order	
Mean (SD)	2.96 (1.88)

**Figure 2:**
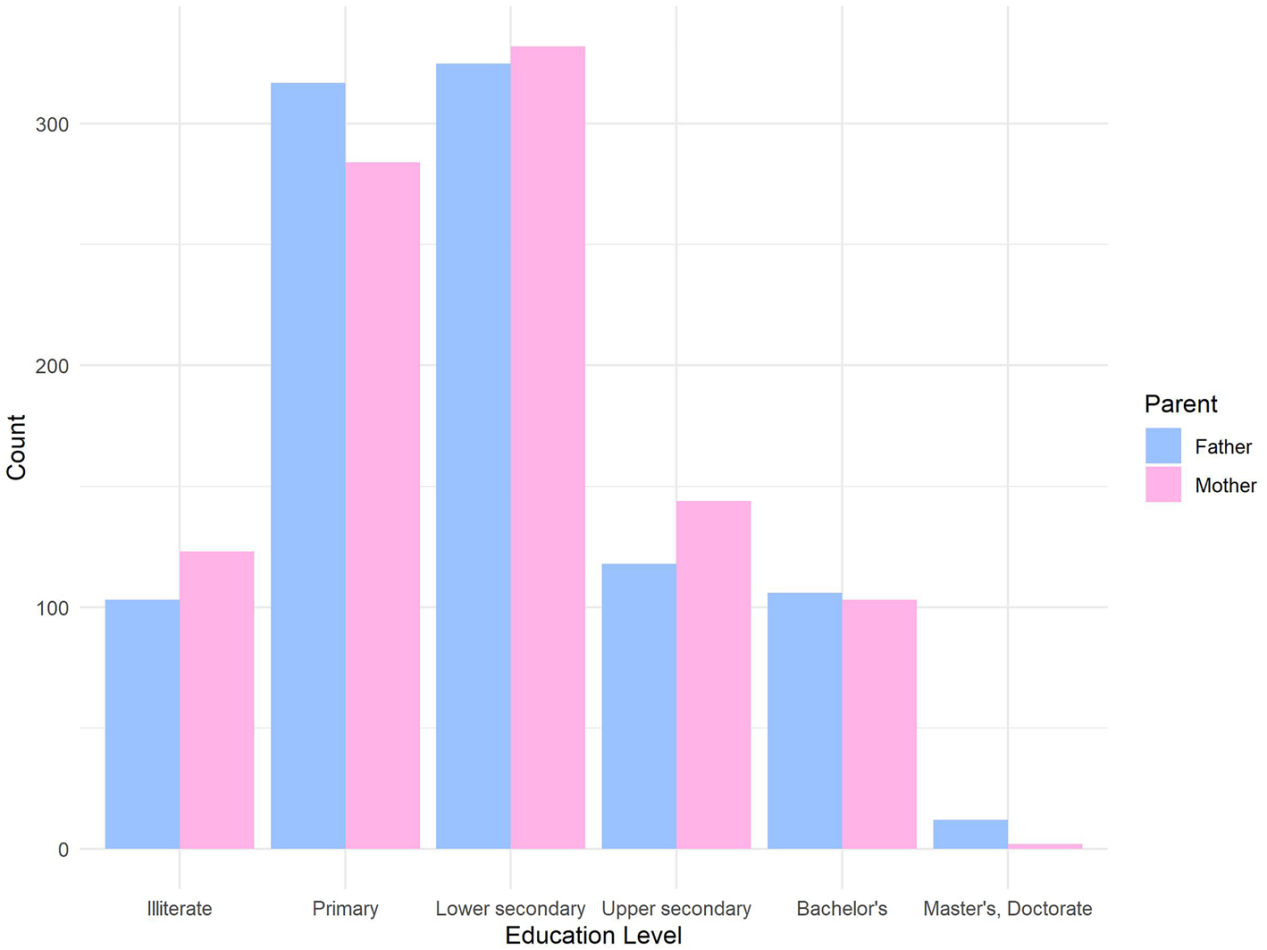
Educational Levels of Parents. Bar chart illustrating the educational levels of children's parents.

The majority of the mothers (89.37%) and the fathers (87.97%) did not pursue a university education or its equivalent. Figure 2 illustrates the educational levels of children parents. A notable 56.28% of the parents expressed that their income was insufficient to meet essential living expenses. Among these families, 35.03% were displaced during the war. Approximately half of the families (55.26%) had their own independent housing. The parents' perception of their overall quality of life was moderate, neither poor nor good (3.06). They reported the highest satisfaction with their social relationships (67.22%) but the lowest satisfaction with their relationships with the environment (43.31%). Table 2 presents an overview of the parents' educational levels, work status, economic status, quality of life (WHOQOL-BREF), and residence status. Our study revealed significant positive correlation (*p*-value < 0.05) between the economic status of the family and the educational level of the parents. Additionally, the overall quality of life of the parents exhibited a positive correlation, particularly with maternal education (*p*-value < 0.05).

**Table 2:** Family status.

Mother’s work status (%)	
Jobless	18 (1.82)
Housewife	838 (84.56)
Farmer	37 (3.73)
Teacher	37 (3.73)
Employee	22 (2.22)
House manager	7 (0.71)
Handcraft	9 (0.91)
Healthcare	11 (1.11)
Retiree	1 (0.10)
Merchant	4 (0.40)
Dead or missed	7 (0.71)
Father’s work status (%)	
Jobless	64 (6.46)
Farmer	64 (6.46)
Teacher	17 (1.72)
Employee	93 (9.38)
House manager	1 (0.10)
Handcraft	429 (43.29)
Healthcare	4 (0.40)
Retiree	15 (1.51)
Merchant	77 (7.77)
Military	118 (11.91)
Driver	49 (4.94)
Student	2 (0.20)
Sport	2 (0.20)
Dead or missed	56 (5.65)
Economic status (%)	
Inadequate to cover necessary living expenses	556 (56.28)
Sufficient to meet necessary living expenses	408 (41.30)
Enough to cover more than just the necessary expenses	24 (2.43)
Displacement during the war (%)	
Yes	345 (35.03)
No	640 (64.97)
Type of their residence (%)	
Separate house	546 (55.26)
With relatives	358 (36.23)
With foreign people	83 (8.40)
Camp	1 (0.10)
Mean of people living in the same household (SD)	
Separate house	6.29 (2.16)
With relatives	11.20 (7.42)
With foreign people	7.47 (4.06)
The mean of the WHOQOL-BREF scores (SD)	
Overall quality of life	3.06 (0.98)
Overall health	3.82 (1.19)
Physical domain %	66.21 (20.84)
Psychological domain %	58.34 (20.65)
Social relationships domain %	67.22 (28.36)
Environment domain %	43.31 (18.61)

27.4% of children in the school-age range 6–17 years were out of school. Furthermore, among the children attending school, 16.67% of them were placed in classes that were two or more grades below their expected level. Table 3 provides a detailed overview of the education status of these children.

**Table 3:** Education of children.

Current grade level for children aged 3–5 years old (%)	
No school	285 (85.08)
Kindergarten	46 (13.73)
First grade	4 (1.19)
Current grade level for children aged 6–17 years old (%)	
No school (never went to school)	103 (17.31)
No school (left school)	60 (10.09)
Kindergarten	8 (1.34)
Primary education (first to sixth grade)	364 (61.18)
Lower secondary education (seventh to ninth grade)	57 (9.57)
Upper secondary education (tenth to twelfth grade)	3 (0.51)
Age-grade discrepancy for children aged 6–17 years old who are currently attending school (%)	
2 years lag	54 (12.50)
3 years lag	9 (2.08)
4 years lag	6 (1.39)
5 years lag	1 (0.23)
6 years lag	2 (0.46)

74.67% of children aged 2–4 years and 61.29% of children aged 4–17 years were experiencing behavioral difficulties. The most prevalent behavioral difficulties observed in both groups were emotional difficulties. Disturbingly, 45.73% of children were exposed to shortages of food and drugs, and almost one-third of children (34.18%) witnessed shooting or bombing incidents. Table 4 highlights the behavioral difficulties experienced by children, along with their exposure to various factors related to war.

**Table 4:** Behavioral difficulties and exposure to war.

Total Difficulties Score for children aged 2–4 years (%)	
Close to average score (0-12)	58 (25.33)
Slightly raised score (13–15)	50 (21.83)
High score (16–18)	47 (20.52)
Very high score (19–40)	74 (32.31)
Prosocial Behavior Score for children aged 2–4 years (%)	
Close to average score (7–10)	181 (79.04)
Slightly lowered score (6)	25 (10.92)
Low score (5)	6 (2.62)
Very low score (0–4)	17 (7.42)
Emotional Behavior Score for children aged 2–4 years (%)	
Within the high and the very high behavioral difficulties range (4–10)	161 (70.31)
Conduct Behavior Score for children aged 2–4 years (%)	
Within the high and the very high behavioral difficulties range (5–10)	98 (42.79)
Hyperactivity Behavior Score for children aged 2–4 years (%)	
Within the high and the very high behavioral difficulties range (7–10)	42 (18.34)
Peer problems Score for children aged 2–4 years (%)	
Within the high and the very high behavioral difficulties range (4–10)	77 (33.62)
Total Difficulties Score for children aged 5–17 years (%)	
Close to average score (0-13)	295 (38.71)
Slightly raised score (14–16)	142 (18.64)
High score (17–19)	139 (18.24)
Very high score (20–40)	186 (24.41)
Prosocial Behavior Score for children aged 5–17 years (%)	
Close to average score (8–10)	583 (76.51)
Slightly lowered score (7)	62 (8.14)
Low score (6)	65 (8.53)
Very low score (0–5)	52 (6.82)
Emotional Behavior Score for children aged 5–17 years (%)	
Within the high and the very high behavioral difficulties range (5–10)	394 (51.71)
Conduct Behavior Score for children aged 5–17 years (%)	
Within the high and the very high behavioral difficulties range (4–10)	308 (40.42)
Hyperactivity Behavior Score for children aged 5–17 years (%)	
Within the high and the very high behavioral difficulties range (8–10)	108 (14.17)
Peer problems Score for children aged 5–17 years (%)	
Within the high and the very high behavioral difficulties range (4–10)	272 (35.7)
Exposure to direct danger (%)	
Yes	216 (21.95)
No	768 (78.05)
Exposure to the shortage of food and drugs (%)	
Yes	450 (45.73)
No	534 (54.27)
Enduring loss within the family (%)	
Yes (due to war)	205 (20.85)
Yes (due to other reasons)	147 (14.95)
No	631 (64.19)
Exposure to Kidnapping (the child or someone else in the family) (%)	
Yes	138 (14.08)
No	842 (85.92)
Witnessing shooting or bombing (%)	
Yes	336 (34.18)
No	647 (65.82)

Children from families with lower economic status displayed higher means of total SDQ scores compared to their peers from higher economic status. Notably, the significant differences persist across economic levels, as confirmed by the Holm–Bonferroni Correction, emphasizing a substantial distinction among various socioeconomic subgroups (*p* value < 0.05) (Table 5). Significant differences were observed in the total SDQ score among children attending school, those who never attended school, and those who had left school. Children who had never attended school presented the highest mean total SDQ score. The Holm-Bonferroni Correction reveals a significant distinction solely between children who never attended school and those currently enrolled, emphasizing the impactful role of educational experiences on behavioral difficulties (*p*-value = 0.011) (Table 5). In terms of residence type, the application of the Holm-Bonferroni Correction highlights a significant difference exclusively in behavioral patterns between individuals residing in separate households and those cohabiting with foreigners (*P*-value = 0.00072). Notably, children living with foreigners exhibit a higher prevalence of behavioral difficulties (Table 5). There was a significant negative correlation between the parents’ age and education levels and the total score of the Strength and Difficulties Questionnaire (SDQ) (Table 6). Significant negative correlations were found between the overall quality of life and the physical, psychological, social, and environmental domains of parents' quality of life and the total SDQ score. Similarly, there were significant negative correlations between the emotional subscale of the SDQ and the parents' overall quality of life, overall health, and each of the mentioned domains. Further correlations between parents' quality of life and children's behavioral difficulties are provided in Table 7.

**Table 5:** Differences of behavioral difficulties between different groups.

Gender	Total SDQ scoreMean ± SD	Total SDQ score	Prosocial score	Prosocial score
		*p*-Value	Mean ± SD	*p*-Value
Female	15.27 ± 5.85	0.05646^b^	8.51 ± 1.79	0.9808^a^
Male	15.95 ± 5.60		8.27 ± 1.91	

Economic status	Total SDQ score	Total SDQ score	Prosocial score	Prosocial score
	Mean ± SD	*p*-Value	Mean ± SD	Pr(> F)
Inadequate to cover necessities	16.07 ± 5.76	**0.0004532**^d,e^	8.40 ± 1.92	0.8083^c^
Sufficient to meet necessities	15.30 ± 5.65		8.36 ± 1.79	
Enough to cover more than necessities	12.08 ± 4.21		8.17 ± 1.76	

Displacement during the war	Total SDQ score	Total SDQ score	Prosocial score	Prosocial score
	Mean ± SD	*p*-Value	Mean ± SD	*p*-Value
Yes	15.80 ± 5.61	0.4296^b^	8.35 ± 1.87	0.3009^a^
No	15.47 ± 5.87		8.42 ± 1.83	

Type of their residence	Total SDQ score	Total SDQ score	Prosocial score	Prosocial score
	Mean ± SD	*p*-Value	Mean ± SD	Pr(> F)
Separate house	15.09 ± 5.60	**0.0002661**^d, f^	8.38 ± 1.82	0.1403^c^
With relatives	16.06 ± 5.87		8.44 ± 1.88	
With foreign people	17.64 ± 5.32		8.11 ± 2.04	
Camp	23.00 ± NA		5.00 ± NA	

School status for 6–17 years old children	Total SDQ score	Total SDQ score	Prosocial score	Prosocial score
	Mean ± SD	*p*-Value	Mean ± SD	*p*-Value
No school (never)	16.83 ± 6.05	**0.02334**^d,g^	8.38 ± 1.88	0.5425^d^
No school (left school)	14.93 ± 6.05		8.32 ± 2.07	
Attending school	15.11 ± 5.83		8.63 ± 1.66	

^a^Welch t-test.

^b^Mann-Whitney test.

^c^One-way analysis of variance (ANOVA).

^d^Kruskal-Wallis test.

^e^Negligible effect size, (the epsilon-squared value 1.579063 × 10^−5^).

^f^Negligible effect size, (the epsilon-squared value 1.954253 × 10^−5^).

^g^Negligible effect size, (the epsilon-squared value 2.483228 × 10^−5^).

Significant values were in bold font.

Total SDQ score, Total Strength and Difficulties Questionnaire score.

**Table 6:** Correlation between multiple variables and the children’s behavioral difficulties.

Variables	r/rho	*p*-Value
Age of mothers versus Total SDQ score	− 0.09^rho^	0.006**
Age of mothers versus prosocial behavior	0.08^r^	0.015*
Age of fathers versus Total SDQ score	− 0.12^rho^	< 0.001***
Age of fathers versus prosocial behavior	0.10^r^	0.003**
Educational level of mothers versus Total SDQ score	− 0.08^rho^	0.010*
Educational level of mothers versus prosocial behavior	0.05^rho^	0.125
Educational level of fathers versus Total SDQ score	− 0.13^rho^	< 0.001***
Educational level of fathers versus prosocial behavior	0.09^rho^	0.004**
Number of siblings versus Total SDQ score	− 0.05^rho^	0.113
Number of siblings versus prosocial behavior	0.04^r^	0.181
Birth order versus Total SDQ score	− 0.04^rho^	0.167
Birth order versus prosocial behavior	0.02^r^	0.469
People living in the same household versus Total SDQ score	0.07^rho^	0.039*
People living in the same household versus prosocial behavior	1.47e-03^r^	0.963
Age-grade discrepancy versus Total SDQ score	0.19^rho^	0.114
Age-grade discrepancy versus prosocial behavior	0.10^rho^	0.386

Significant values were in bold font. r is for Pearson correlation, and rho is for Spearman correlation, * P ≤ 0.05, ** P ≤ 0.01, *** P ≤ 0.001.

Total SDQ score, Total Strength and Difficulties Questionnaire score.

**Table 7:** Correlation between the parents’ quality of life and the children’s behavioral difficulties.

Variables	r/rho	*p*-Value
Overall quality of life versus Total SDQ score	− 0.07^rho^	0.022*
Overall quality of life versus prosocial behavior	0.02^r^	0.631
Overall quality of life versus conduct problems	− 8.93e-03^r^	0.779
Overall quality of life versus hyperactivity behavior	2.71e-03^r^	0.932
Overall quality of life versus emotional symptoms	− 0.14^r^	< 0.001***
Overall quality of life versus peer problems	-0.04^r^	0.205
Overall health versus Total SDQ score	− 0.05^rho^	0.127
Overall health versus prosocial behavior	0.06^r^	0.056
Overall health versus conduct problems	− 3.39e-03^r^	0.915
Overall health versus hyperactivity behavior	− 0.01^r^	0.721
Overall health versus emotional symptoms	− 0.12^r^	< 0.001***
Overall health versus peer problems	− 0.04^r^	0.185
Physical domain versus Total SDQ score	− 0.12^rho^	< 0.001***
Physical domain versus prosocial behavior	0.07^r^	0.034*
Physical domain versus conduct problems	− 0.02^r^	0.468
Physical domain versus hyperactivity behavior	− 0.08^r^	0.011*
Physical domain versus emotional symptoms	− 0.19^r^	< 0.001***
Physical domain versus peer problems	− 0.06^r^	0.041*
Psychological domain versus Total SDQ score	− 0.12^rho^	< 0.001***
Psychological domain versus prosocial behavior	0.04^r^	0.270
Psychological domain versus conduct problems	− 0.08^r^	0.014*
Psychological domain versus hyperactivity behavior	− 0.09^r^	0.005**
Psychological domain versus emotional symptoms	− 0.13^r^	< 0.001***
Psychological domain versus peer problems	− 0.04^r^	0.227
Environment domain versus Total SDQ score	− 0.16^rho^	< 0.001***
Environment domain versus prosocial behavior	− 0.01^rho^	0.711
Environment domain versus conduct problems	− 0.07^rho^	0.025*
Environment domain versus hyperactivity behavior	− 0.06^rho^	0.083
Environment domain versus emotional symptoms	− 0.19^rho^	< 0.001***
Environment domain versus peer problems	− 0.08^rho^	0.009**
Social relationships domain versus Total SDQ score	− 0.10^rho^	0.003**
Social relationships domain versus prosocial behavior	0.07^r^	0.037*
Social relationships domain versus conduct problems	− 0.06^r^	0.065
Social relationships domain versus hyperactivity behavior	− 0.03^r^	0.306
Social relationships domain versus emotional symptoms	− 0.07^r^	0.031*
Social relationships domain versus peer problems	− 0.12^r^	< 0.001***

Significant values were in bold font. r is for Pearson correlation, and rho is for Spearman correlation, * P ≤ 0.05, ** P ≤ 0.01, *** P ≤ 0.001.

Total SDQ score, Total Strength and Difficulties Questionnaire score.

**Figure 3:**
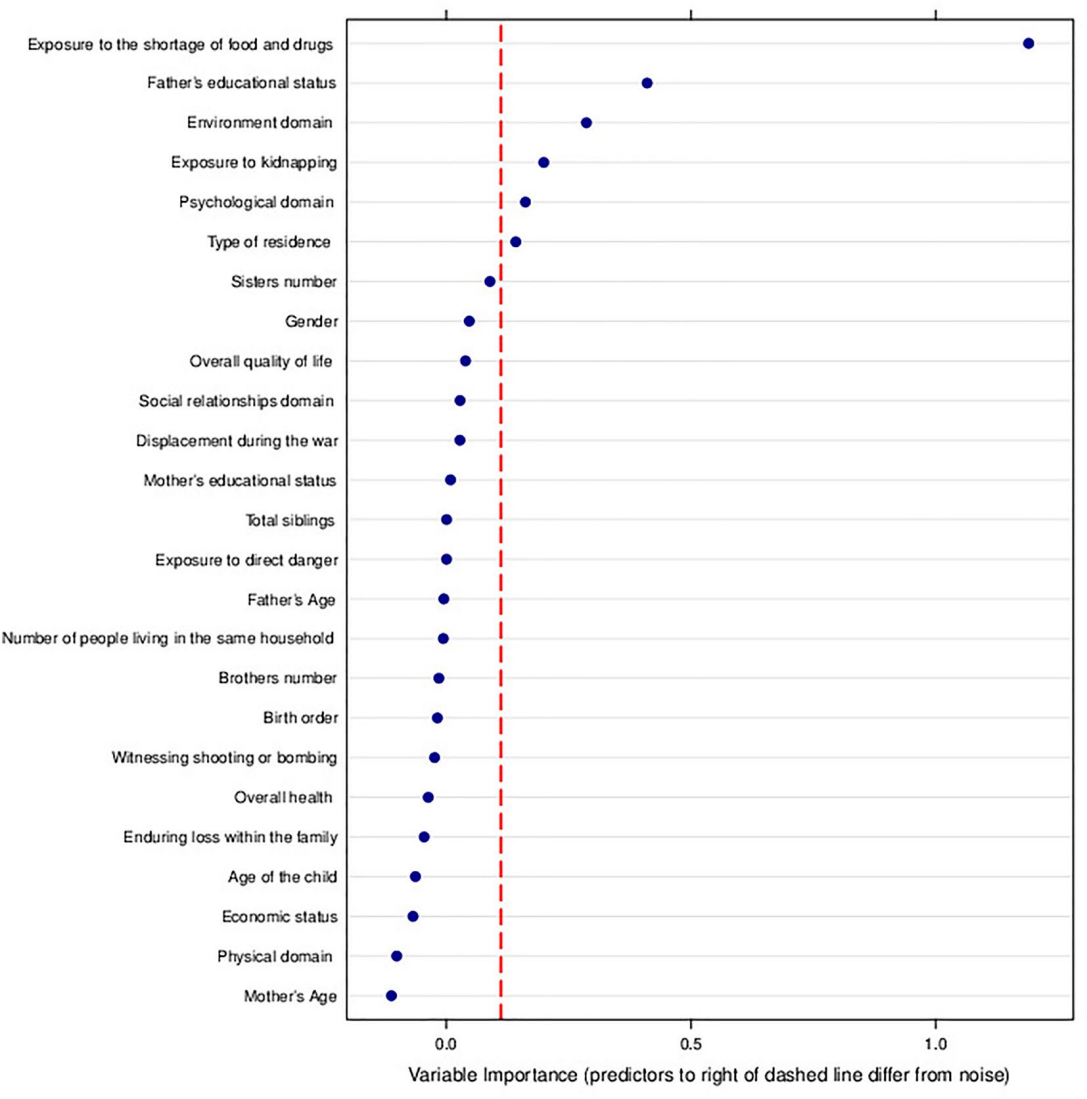
Conditional Random Forest Analysis for Children Aged 2–4 Years. The dashed red line indicates separation for important variables; those to the right are unlikely random noise.

**Figure 4:**
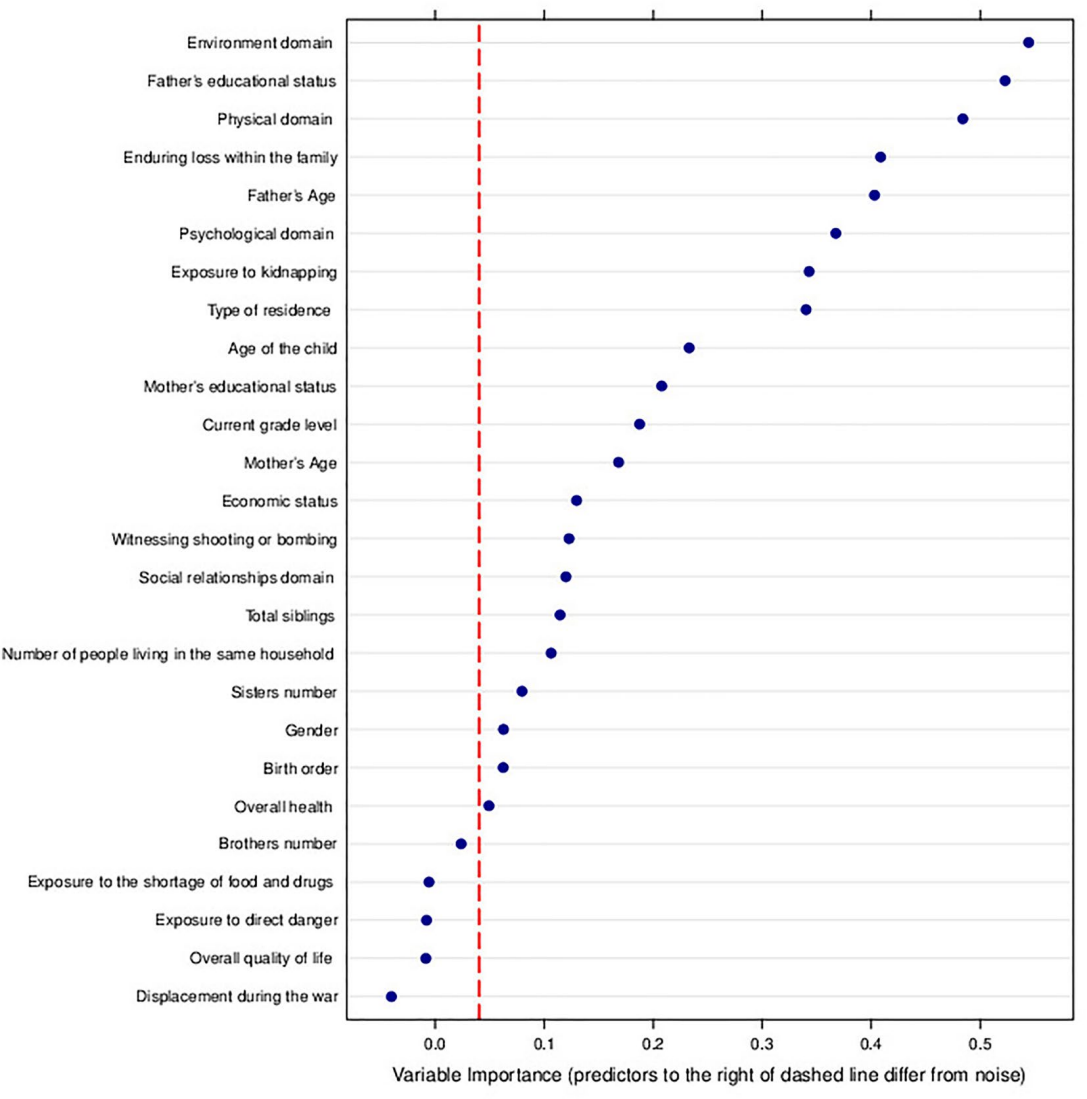
Conditional Random Forest Analysis for Children Aged 5–17 Years. The dashed red line indicates separation for important variables; those to the right are unlikely random noise.

The total SDQ score showed significant differences between children who were exposed to kidnapping and those who were not. The estimated effect size (Cliff's Delta) for [Kidnapping] is 0.9997, indicating a large effect. The 95% confidence interval (CI 0.9989–0.9999) is notably narrow, providing a high level of precision in estimating the magnitude of the observed effect. Children who endured the loss of family members due to the war had the highest mean total SDQ scores. Findings reveal a significant difference in children's behavior (total score and Prosocial score). Notably, the Holm–Bonferroni Correction emphasizes that the significant distinction is observed solely between children who have not experienced any loss of family members and those who have encountered loss solely due to the war. Moreover, emotional difficulties were significantly higher in children exposed to direct danger, kidnapping, and loss of a family member. Table 8 illustrates the total SDQ scores and prosocial behavior among children exposed to different war-related factors. Taking into account various covariates, including economic status, gender, type of residence, maternal and paternal education, maternal and paternal age, number of people living in the house, overall health of the parents, and all parameters related to the quality of life of the parents, the relationship between family loss, kidnapping, and children’s behavior remains statistically significant, highlighting the robustness and complexity of these associations (*p* value < 0.05). In our study we analyzed the complex relationship between war-related variables and family factors to identify the foremost predictor of children's behavior within the 2–4 age range using random forest analysis. Our findings revealed that exposure to a shortage of food and drugs emerged as the most influential predictor. Other noteworthy predictors, in order of significance, include the educational level of fathers, the environmental domain, exposure to kidnapping, the psychological domain of parents’ quality of life, and the type of residence (Fig. 3). Using the random forest analysis for children and adolescents aged between 5 and 17 years old, our study identified the environmental quality of life, educational level of fathers, physical quality of life, psychological quality of life, and enduring loss within the family as the most influential predictors. Additional noteworthy predictors are illustrated in Fig. 4.

**Table 8:** Differences of behavioral difficulties between groups with different exposure to war.

	Total SDQ score	Total SDQ score	Prosocial score		Prosocial score
	Mean ± SD	*p*-Value	Mean ± SD		*p*-Value
Exposure to direct danger					
Yes	15.90 ± 6.14	0.4261^b^	8.36 ± 1.87		0.4188^a^
No	15.58 ± 5.60		8.39 ± 1.85		
Exposure to the shortage of food and drugs					
Yes	16.01 ± 6.04	0.07068^b^		8.51 ± 1.85	0.9757^a^
No	15.34 ± 5.41		8.28 ± 1.85		
Exposure to kidnapping					
Yes	17.54 ± 5.91	**6.078e-05**^b^		8.34 ± 1.85	0.3764^a^
No	15.34 ± 5.64		8.39 ± 1.85		
Witnessing shooting or bombing					
Yes	15.67 ± 6.35	0.9572^b^		8.53 ± 1.77	0.9683^a^
No	15.65 ± 5.36		8.31 ± 1.89		
Enduring loss within the family					
Yes (due to war)	16.74 ± 6.13	**0.001473**^d,e^		8.41 ± 1.80	**0.007466**^d^
Yes (other reasons)	16.15 ± 5.59			8.84 ± 1.47	
No	15.19 ± 5.56		8.27 ± 1.94		

^a^Welch t-test.

^b^Mann-Whitney test.

^c^One-way analysis of variance (ANOVA).

^d^Kruskal-Wallis test.

^e^Negligible effect size, (the epsilon-squared value 1.674396 × 10^−5^).

Significant values were in bold font.

Total SDQ score, Total Strength and Difficulties Questionnaire score.

## Discussion

Previous research conducted within Syria primarily focused on the prevalence of post-traumatic stress disorder (PTSD) in Syrian children, which was relatively high among school children, ranging between 53 and 90%^39,40^. However, to our knowledge, no published studies have addressed the prevalence of emotional and behavioral issues among Syrian children and adolescents within the country. In this study, it was found that over two-thirds of the children and adolescents living in Syria were experiencing behavioral difficulties, with the emotional being the most common difficulties. This prevalence was higher compared to Syrian refugee children in neighboring countries, where only 25–31% of them experienced behavioral problems^21,41^. The higher prevalence of behavioral difficulties among children in Syria, as compared to their refugee peers, can be attributed to the removal of refugee children from the war environment in Syria. However, both Syrian children inside and outside the country experienced more emotional and behavioral difficulties compared to children raised in other developing^42–49^ or developed countries^50–53^. The Syrian crisis, considered the most severe humanitarian catastrophe of the 21st century^8^, is characterized by ongoing violence and destruction^9^. Syrian children have experienced loss, injuries, disrupted education, and witnessed violence^54^, leading to "toxic stress" with severe long-term psychological and physical consequences^55,56^. Nearly half of the children in this study faced shortages of food and medications, while almost one-third witnessed shooting or bombing incidents. Children who experienced kidnapping or the loss of family members due to the war exhibited significantly higher levels of behavioral difficulties, particularly emotional distress. A similar trend was observed among Syrian child refugees, where 45.6% of those exposed to war traumas developed PTSD, which may be linked to emotional dysregulation^57–59^.

Furthermore, the high prevalence of behavioral difficulties stems not only from the direct impacts of the war on individual children but also from its consequences within their immediate and broader environments. The ongoing Syrian conflict significantly impacts all aspects of Syrian life, thereby undermining children’s family and education, two foundational pillars of their well-being and future^26,54^. The majority of parents in the study have limited education. This can be traced back to 11 years ago when roughly one-third of the mothers in the study were 17 years old or younger, and nearly half of the parents were aged 18–28. The impact of the war during this period likely disrupted their education and, for the older group, hindered their access to higher education. We found that children with less-educated parents tend to display more behavioral difficulties, which aligns with previous studies^22,43,52,60^. Importantly, our study highlighted that the educational level of fathers stands out as one of the influential predictors for children’s behavior. Parental and maternal educational levels can shape children's behavior^61^. For instance, the association between hyperactivity symptoms is linked to parental education rather than shared familial and genetic risk factors^62^. The impact of lower educational levels on child development can be attributed to well-educated parents' awareness of their child's development. They can employ suitable approaches to support their children's growth and seek appropriate resources when issues arise^63^. Our study aligns with existing literature, indicating that families with lower educational levels tend to have lower income and socioeconomic status. We found a positive and significant correlation between maternal and paternal education and the economic status of the family^22^. Over half of our sample had insufficient income to cover essential living expenses, and the worse the economic situation of parents, the more pronounced their children’s behavioral difficulties. This link between low socioeconomic status and children's behavior is well-documented in existing literature^43,60,64^. Family income directly affects children's behavior by limiting access to healthcare and essentials^63,65^, and indirectly by causing parental distress and impairing parenting^60,65,66^. Limited parents' education and lower economic status impact children’s education^62,67^. Notably, 27.4% of school-age children 6–17 years were not enrolled in school. Additionally, children who had never attended school had the highest behavioral difficulties. This link can be attributed to the positive influence of peer relationships on children's development^68^. The presence of values education and school counselors is associated with improved child behavior^69,70^.

Parents rated their overall quality of life as moderate, reporting the highest satisfaction with their social relationships but the lowest with their environment and mental well-being. This result may not fully reflect parents’ true sentiments due to cultural norms in Syria favoring positive responses about one’s situation. When parents reported a lower quality of life considering all aspects, it was associated with behavioral difficulties in the children, especially emotional difficulties. This association between caregiver and child mental health remains consistent in both conflict and non-conflict settings, although the exact mechanisms behind this connection are not fully understood. Research has suggested that children exposed to their parents’ trauma or mental illness may display irritability, or outbursts of anger. These behavioral issues in children may, in turn, lead to more frequent physical punishment by traumatized parents^71^. These difficulties in parent–child interactions can result in reduced monitoring, decreased family cohesion, and increased conflict. However, not all children with traumatized parents exhibit these impairments; some display resilience. Their resilience is likely bolstered by their perception of a supportive family environment despite their parents' challenges and their positive relationships with peers. This underscores the significance of a supportive environment in enhancing children's well-being^72^.

## Limitations

Our study encountered challenges related to the distinctive context of the children's hospital where patient visits occur spontaneously without prior appointment registration. This context posed significant obstacles to the application of random sampling techniques, as patient attendance was not systematically controlled. Additionally, a notable limitation of our study is the reliance on a clinic-based sample of children and adolescents, as opposed to a school-based one. This choice may introduce a potential bias, as the clinic sample may not fully represent the entire population. However, around 27% of our data comes from individuals not in school, so focusing exclusively on schools would have missed this group. Future studies could use a dual-sample approach, including both school and clinic samples for more comprehensive insights.

## Conclusion

The novelty of this study stems from being the first to examine behavioral difficulties in Syrian children within the country, addressing the direct and indirect impact of the ongoing war. This study's conclusion is sobering: our Syrian nation is suffering, with highly prevalent behavioral and emotional difficulties among children and adolescents. These difficulties aren't solely caused by direct war-related factors like kidnappings and family losses; they also result from broader, indirect effects impacting children's home environments, education, and their parents’ quality of life. The implications of these challenges, coupled with the fact that almost a third of our children are not attending school, extend beyond the children's current lives and affect future generations, trapping them in a cycle of trauma and dysfunction. Urgent efforts are needed to halt the war and its devastating impact on our last hope—the children—to secure the future of our nation.

## Data Availability

The data can be made available upon reasonable request from the corresponding author.

## Abbreviations

ANOVA: One-way analysis of variance
ANCOVA: Analysis of covariance
PTSD: Post-traumatic stress disorder
SDQ: Strengths & difficulties questionnaire
WHOQOL-BREF: The brief Arabic version of the World Health Organization quality of life questionnaire
